# T2-sparing vs T2-including sympathectomy for hyperhidrosis: a meta-analysis on compensatory sweating

**DOI:** 10.1186/s13019-026-04464-4

**Published:** 2026-06-25

**Authors:** Lucas Monteiro Delgado, Paula Duarte D’Ambrosio, Rachid Eduardo Noleto da Nobrega Oliveira, Keith Mortman, Túlio Caldonazo, Felipe S. Passos, Marcelo Albuquerque Barbosa Martins, Deepti Sreepathi, Adriana-Simoneta Pimienta-Ibarra, Paulo Manuel-Pêgo Fernandes, Nelson Wolosker, José Ribas Milanez Campos

**Affiliations:** 1 Federal University of Minas Gerais, Belo Horizonte, Minas Gerais Brazil; 2 Department of Thoracic Surgery, Faculdade de Medicina, Hospital das Clinicas de Sao Paulo, Universidade de Sao Paulo, Sao Paulo, Brazil; 3 Department of Thoracic Surgery, Barretos Cancer Center, São Paulo, Brazil; 4 Division of Thoracic Surgery, The George Washington University, Washington, D.C., USA; 5 Department of Cardiothoracic Surgery, Jena University Hospital, Am Klinikum 1, 07747 Jena, Germany; 6 Department of Thoracic Surgery, MaterDei Hospital, Salvador, Brazil; 7 Federal University Ouro Preto, Ouro Preto, Minas Gerais Brazil; 8 Independent Scholar, Boston, MA USA; 9 Department of Thoracic Surgery, Instituto Nacional de Enfermedades Respiratorias Ismael Cosío Villegas, Mexico City, Mexico; 10 Department of Thoracic Surgery, Faculdade de Medicina, Hospital das Clinicas de Sao Paulo, Universidade de Sao Paulo, Sao Paulo, Brazil; 11 Department of Health Sciences, Hospital Israelita Albert Einstein, Sao Paulo, Brazil; 12 Department of Vascular and Endovascular Surgery, Faculdade de Medicina, Universidade de São Paulo, São Paulo, SP Brazil

**Keywords:** Compensatory sweating, Endoscopic thoracic sympathectomy, Meta-analysis, Hyperhidrosis

## Abstract

**Background:**

Sympathectomy is the definitive treatment for primary hyperhidrosis, offering high success rates. However, compensatory sweating (CS) remains a frequent and distressing complication. The level of ganglionic resection, particularly the inclusion of T2, may influence CS incidence, but evidence remains inconsistent.

**Methods:**

A systematic search of PubMed, Embase, and the Cochrane Library was conducted up to July 2025, to identify studies comparing T2-sparing versus T2-including sympathectomy for primary hyperhidrosis. Pooled odds ratios (ORs) and 95% confidence intervals (CIs) were calculated using random-effects models. Heterogeneity was assessed using the I2 statistic. For outcomes with significant heterogeneity, leave-one-out sensitivity analyses were performed.

**Results:**

Eleven studies involving 3,090 patients were included. Of these, 47.2% underwent T2-sparing sympathectomy and 52.8% underwent T2-including procedures. T2-sparing sympathectomy was associated with a significantly lower incidence of overall CS (OR 0.38; 95% CI 0.21–0.67; p = 0.0009; I2 = 65%) and severe CS (OR 0.43; 95% CI 0.28–0.64; p < 0.0001; I2 = 19%). Subgroup analyses confirmed consistent results across both randomized and non-randomized studies, as well as across short-term (≤ 12 months) and long-term (> 12 months) follow-up periods. Sensitivity analyses confirmed the robustness of the findings. No publication bias was detected.

**Conclusions:**

T2-sparing sympathectomy was associated with a significantly lower incidence of CS. These findings support avoiding T2 when feasible to minimize postoperative morbidity. Further prospective studies are needed to confirm these results.

**Supplementary Information:**

The online version contains supplementary material available at 10.1186/s13019-026-04464-4.

## Introduction

Primary hyperhidrosis is a condition characterized by excessive sweating in specific areas, most commonly affecting the palms, armpits, and feet. It is a relatively common disorder, with an estimated population prevalence of approximately 1–5%, typically beginning during childhood or adolescence and persisting into adulthood. It affects both sexes and frequently impairs quality of life and daily functioning [2]. While there are non-surgical treatments available, endoscopic thoracic sympathectomy (ETS) is considered the gold standard for definitive management in severe or refractory cases [1]. This procedure has a high success rate and can lead to substantial improvements in quality of life [2].

Despite the effectiveness of ETS, compensatory sweating (CS) is the most frequent and troublesome postoperative complication, with reported incidence rates ranging from 3 to 98% [3]. CS typically affects previously unaffected regions, such as the abdomen, chest, thighs, or back, and can range from mild discomfort to severe debilitating sweating [4, 5]. While some patients tolerate CS in exchange for symptom relief, others report significant dissatisfaction and regret following the surgery.

The level of sympathetic chain interruption has been proposed as a potential modifiable factor influencing the incidence of CS. In particular, resections involving the T2 ganglion have been associated with an increase in CS in some observational studies, although these findings do not always reach statistical significance [6, 7]. Conversely, other analyses suggest that restricting the resection to lower levels (e.g., T3–T4) may not significantly reduce the incidence of CS [8]. A recent meta-analysis conducted by Lin and Lin identified factors such as age, body mass index, and smoking as risk factors for CS, but did not evaluate the impact of resection level, specifically the inclusion of the T2 ganglion, which is an important surgical variable [9].

This systematic review and meta-analysis aim to assess whether including the T2 ganglion in ETS is associated with a higher incidence of CS compared to approaches that spare the T2 ganglion. The goal is to inform surgical techniques and improve patient outcomes.

## Methods

We performed a systematic review and meta-analysis following the guidelines outlined in the Cochrane Handbook for Systematic Reviews of Interventions. The structure of the review aligns with the Preferred Reporting Items for Systematic Reviews and Meta-Analysis (PRISMA) guidelines [10, 11]. Additionally, the study protocol was registered in the International Prospective Register of Systematic Reviews (PROSPERO) under registration number CRD420251108326 [12].

### Outcomes and subgroup analysis

The outcomes assessed included: (1) any CS at any follow-up time point, generally defined as new postoperative sweating in body regions not primarily affected before sympathectomy; and (2) severe CS at any follow-up time point, broadly defined as compensatory sweating associated with substantial symptom burden, such as social embarrassment, intolerance, or interference with daily activities. Given the variability in definitions, grading systems, and assessment approaches across studies, detailed study-specific criteria and related methodological features are summarized in Supplementary Table I.

We conducted subgroup analyses based on study design (Randomized Controlled Trials [RCTs] vs non-RCTs), follow-up duration (≤ 12 months vs > 12 months), and sympathectomy technique (cautery vs clipping). We also performed exploratory subgroup analyses restricted to studies with the same number of interrupted levels in both arms, as well as to studies in which the T2-sparing group involved fewer interrupted levels than the control group.

### Eligibility criteria

Inclusion in this meta-analysis was restricted to studies that met all of the following eligibility criteria: (1) enrollment of patients with primary hyperhidrosis; (2) RCTs and observational studies; (3) comparison of T2-included versus T2-sparing sympathectomy; (4) reporting on at least one outcome of interest.

Studies were excluded based on the following criteria: (1) studies without a comparative arm; (2) those using techniques other than surgical procedures; (3) unavailable full text; (4) publications classified as case reports, trial registrations with no available results, meta-analyses, reviews, or animal studies.

### Search strategy and study selection

We systematically searched PubMed, Embase, and Cochrane Library databases from inception to July 13th, 2025. The search strategy included the following terms: ("hyperhidrosis" OR "primary hyperhidrosis") AND ("sympathectomy" OR "thoracic sympathectomy" OR "endoscopic thoracic sympathectomy" OR "ETS") AND ("compensatory sweating" OR "compensatory hyperhidrosis") AND ("T2" OR "T3" OR "T4" OR "T5" OR "level of resection" OR "sympathetic level").

Two authors (P.D.D. and A.S.P.I) independently conducted the search, imported results into Rayyan, a web-based systematic review tool, and triaged the studies. After excluding duplicates and titles/abstracts unrelated to the clinical question, the eligibility of each remaining study was assessed through a review of the full-text articles. Disagreements were solved by consensus.

### Data extraction

Two authors (L.M.D. and M.A.B.M) independently extracted data from the included studies using a standardized form. The extracted information included: general study details (title, authors, year of publication, study design, country, the interventions and control groups adopted, and the hyperhidrosis distribution), number of participants, patient characteristics (age, sex, and family history), follow-up duration, and reported outcomes.

### Risk of bias assessment

The risk of bias was assessed using the Cochrane Risk of Bias tool for randomized trials (RoB 2) and the Risk of Bias In Non-randomized Studies of Interventions (ROBINS-I V2) tool for observational studies [13, 14]. Two independent authors conducted the risk of bias assessment (A.S.P.I and P.D.D.) and disagreements were solved by consensus. Publication bias was assessed through visual inspection of contour-enhanced funnel plots and statistical assessment using Egger’s regression asymmetry test and Begg’s rank correlation test [11, 15, 16].

### Statistical analysis

We used the DerSimonian-Laird random-effects model to calculate pooled odds ratios (ORs) for dichotomous outcomes with their respective 95% confidence intervals (CIs) [17]. Significance was regarded as a *p*-value < 0.05. Between‐study heterogeneity was assessed using the Cochran Q test and I^2^ statistics, and we considered a P value of < 0.10 and an I^2^ > 25% as significant for heterogeneity. For outcomes with significant heterogeneity, we performed leave-one-out sensitivity analysis to identify influential studies and their effect on the pooled estimates. We conducted all the statistical analyses using R statistical software (version 4.3.3; R Foundation for Statistical Computing, Vienna, Austria).

## Results

### Study selection and characteristics

**Fig. 1:**
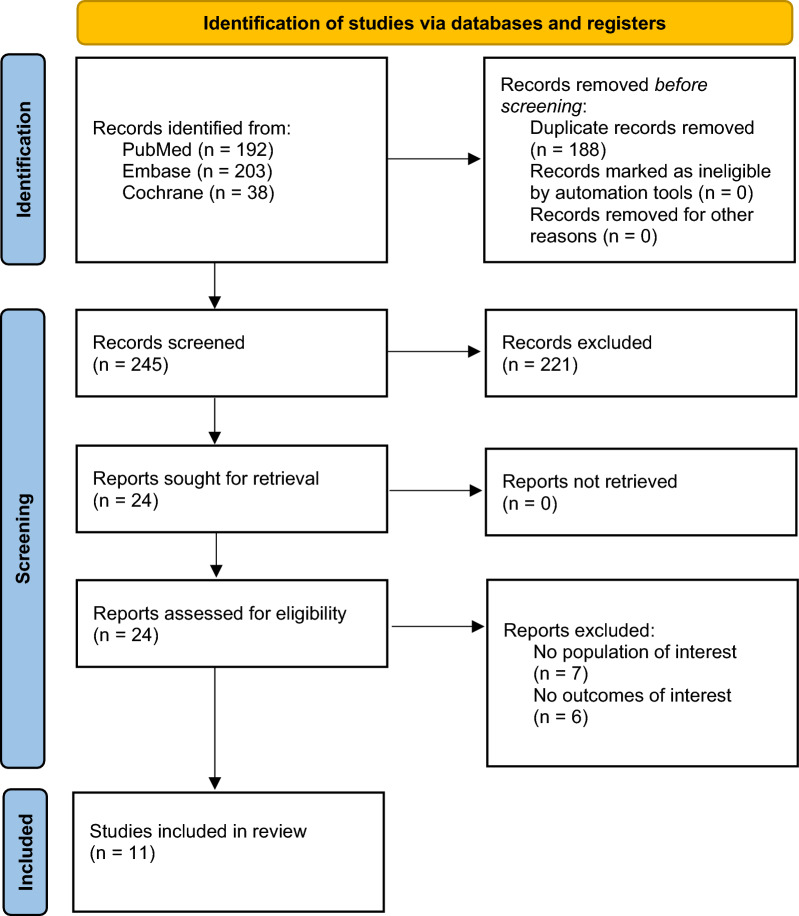
PRISMA flow diagram. Flow diagram summarizing the number of records identified, screened, excluded, and included in the systematic review and meta-analysis of thoracic sympathectomy for primary hyperhidrosis. PRISMA indicates Preferred Reporting Items for Systematic Reviews and Meta-Analyses

As detailed in Fig. 1, the initial search yielded 433 results. After removing duplicate records and screening titles and abstracts, 24 studies were selected for full-text review based on the predefined criteria. Of these, 11 studies met the eligibility requirements (4 RCTs; 5 retrospective cohorts; 2 prospective cohorts) and were included in the analysis, involving a total of 3,090 patients [18–28]. Among these patients, 1,460 (47.2%) underwent T2-sparing sympathectomy, while 1,630 (52.8%) underwent T2-included sympathectomy. The overall proportion of male patients was approximately 52.1%. The reported mean or median age across studies ranged from 20.4 to 34.7 years. Where available, a family history of hyperhidrosis was reported in about 41.5% of the patients. The main characteristics of the included studies are summarized in Table 1.

**Table 1:** Baseline characteristics of the included studies

**First author, year**	**Country**	**Design**	**Period**	**Intervention**	**Control**	**Hyperhidrosis type**	**Technique**	**No. of patients**		**Male, n (%)**		**Mean age, years**		**Family history, %**			**Mean follow-up, months**	
								**T2 Sparring**	**T2 Including**	**T2 Sparring**	**T2 Including**	**T2 Sparring**	**T2 Including**	**T2 Sparring**	**T2 Including**			
Cai, 2014	China, single-center	RCT	2008–2010	T3-T4	T2-T4	Palmar	Cautery	60	56	33 (55)	24 (42.9)	25.8	24.6	23.2	21.4	12†⸸		
Chang, 2007	Taiwan, single-center	R-Obs	2000–2004	T3-T4	T2	Palmar	Cautery	148	86	73 (49)	41 (48)	25.0	20.4	48.2†		39.2		60.9
Ersin, 2024	Taiwan, single-center	R-Obs	2018–2021	T3-T5	T2-T4	Palmar, Axillary and Plantar	Cautery	62	88	30 (48.4)	48 (54.6)	22.5*	26*	100†		4–77†☨		
Esme, 2019	Turkey, single-center	R-Obs	2011–2017	T3–T4	T2–T3	Palmar, Axillary and Plantar	Cautery	125	29	88 (70.4)	17 (58.6)	24†		NA	NA	13†		
Reisfeld, 2007	United States, single-center	R-Obs	1999–2004	T3-T4	T2-T3	Palmar, Axillary, Plantar and Facial	Clipping	656	618	312 (47.6)	336 (54.5)	29.0	29.4	47.7	43.7	8.2		17.9
Salim, 2018	Egypt, single-center	RCT	2012–2016	T3-T4	T2-T4	Palmar and Axillary	Cautery	60	60	31 (51.7)	34 (56.7)	21.2	21.4	51.7	55	12†⸸		
Schmidt, 2006	Germany, single-center	P-Obs	2000–2003	T3-T5	T2-T4	Palmar, Axillary, Plantar and Facial	Cautery	61	117	9 (14.8)	42 (35.9)	34.7	32.1	NA	NA	44.3†		
Scognamillo, 2011	Italy, single-center	R-Obs	1993–2007	T3-T4	T2-T4	Palmar and Axillary	Cautery + Clipping	21	67	NA	NA	NA	NA	NA	NA	1–180☨		
Sugimura, 2009	Canada, single-center	P-Obs	1998–2006	T3-T4	T2-T3	Palmar, Axillary, and Facial	Clipping	212	454	383 (53)†		26.9*†		NA	NA	10.4*†		
Turkyilmaz, 2017	Turkey, single-center	RCT	2014–2015	T3	T2	Palmar	Cautery	25	25	15 (60)	18 (72)	22.8	21.8	NA	NA	13.6*		13.0*
Yazbek, 2009	Brazil, single-center	RCT	2003–2006	T3	T2	Palmar	Cautery	30	30	19 (31.7)†		23.2	23.4	a		20⸸		

^*^median; † data from both groups combined; ☨ range; ⸸maximum follow-up reported. a: more than half of the included patients had positive family history of hyperhydrosis; NA: Not available; P-Obs: Prospective observational study; R-Obs: Retrospective observational study; RCT: Randomized controlled trial

### Pooled analyses

#### Compensatory sweating

**Fig. 2:**
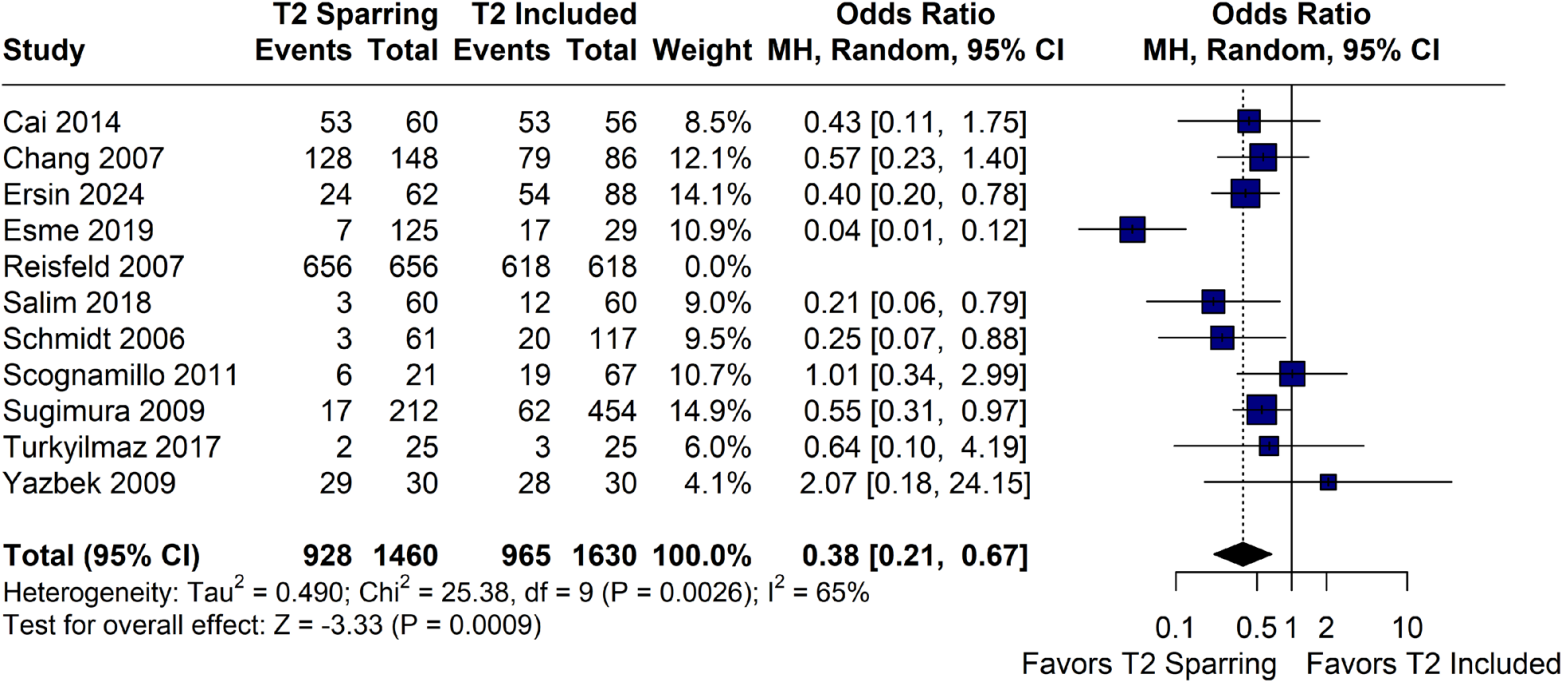
Forest plot of overall compensatory sweating. Forest plot comparing the occurrence of overall compensatory sweating between T2-sparing and T2-including sympathectomy, stratified by study design. Squares represent individual study effect estimates, with square size proportional to study weight. Horizontal lines indicate 95% confidence intervals. Diamonds represent pooled estimates. CI indicates confidence interval; MH, Mantel–Haenszel method

Overall CS (n = 3,090; OR 0.38; 95% CI 0.21–0.67; p = 0.0009; I2 = 65%; Fig. 2) was significantly lower in the T2-sparing group.

#### Severe compensatory sweating

**Fig. 3:**
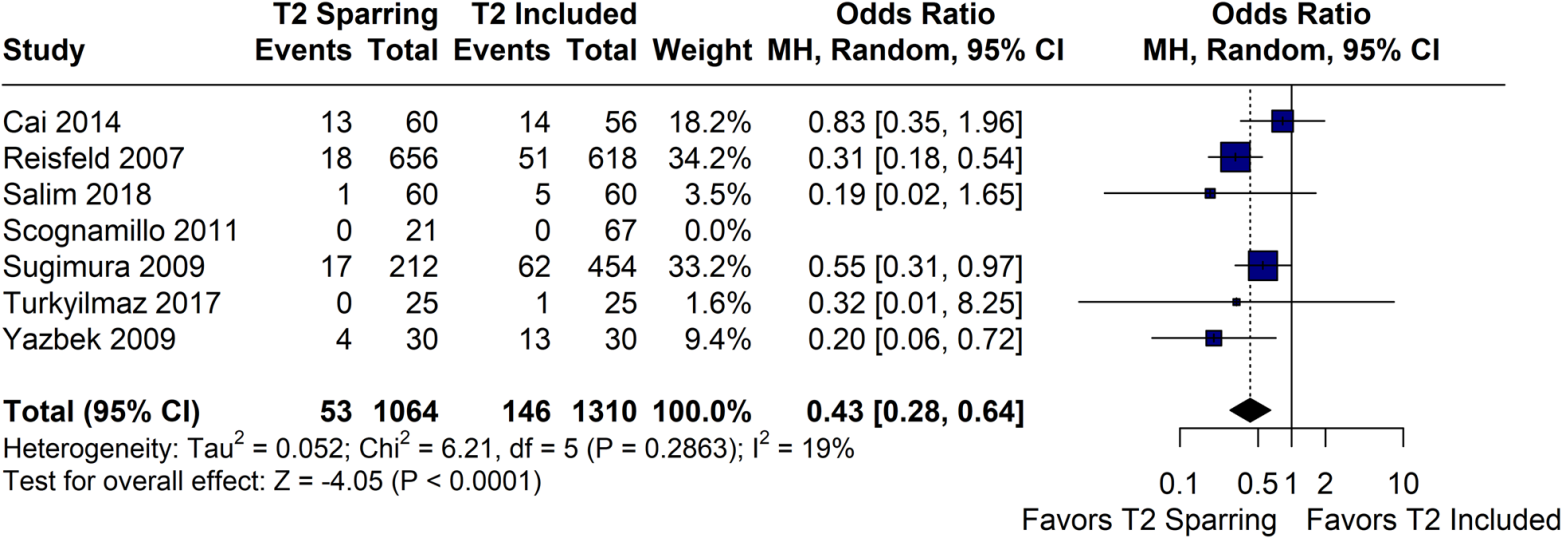
Forest plot of severe compensatory sweating. Forest plot comparing the occurrence of severe compensatory sweating between T2-sparing and T2-including sympathectomy, stratified by study design. Squares represent individual study effect estimates, with square size proportional to study weight. Horizontal lines indicate 95% confidence intervals. Diamonds represent pooled estimates. CI indicates confidence interval; MH, Mantel–Haenszel method

Severe CS (n = 2,374; OR 0.43; 95% CI 0.28–0.64; p < 0.0001; I2 = 19%; Fig. 3) was also significantly lower in the T2-sparing group.

### Subgroup analysis

#### Compensatory sweating

In the subgroup analysis by study design, the reduction in CS with T2-sparing sympathectomy was observed in both observational studies (n = 2,744; OR 0.34; 95% CI 0.16–0.73; I2 = 78%) and RCTs (n = 346; OR 0.42; 95% CI 0.19–0.94; I2 = 0%), with no significant difference between subgroups (p = 0.7263), as shown in Supplementary Figure S2.

In the subgroup analysis by follow-up duration, the reduction in CS associated with T2-sparing sympathectomy was observed at both short-term follow-up (≤ 12 months; OR 0.47; 95% CI 0.29–0.76; I^2^ = 0%) and long-term follow-up (> 12 months; OR 0.38; 95% CI 0.16–0.89; I^2^ = 74%). There was no significant difference between these subgroups (p = 0.6780), indicating that the benefit of T2-sparing sympathectomy persists over time, as shown in Supplementary Figure S3. The study by Reisfeld et al. [22] was excluded from this analysis due to a substantial difference in follow-up duration between the groups.

In the subgroup analysis by surgical technique, the reduction in CS associated with T2-sparing sympathectomy was observed in both the cautery subgroup (OR 0.31; 95% CI 0.15–0.63; I2 = 64%) and the clipping subgroup (OR 0.55; 95% CI 0.31–0.97). There was no significant difference between these subgroups (p = 0.2070), as shown in Supplementary Figure S4. The study by Scognamillo et al. [25] was not included in this analysis because the T2-included arm consisted of sympathectomy, whereas the T2-sparing arm was mixed, including both sympathicotomy and ganglion block by clipping.

In the exploratory subgroup analysis based on denervation extent, the reduction in CS associated with T2-sparing sympathectomy was observed in studies with the same number of interrupted levels in both arms (OR 0.32; 95% CI 0.13–0.77; I2 = 76%; Supplementary Figure S5). In studies in which the T2-sparing group involved fewer interrupted levels than the control group, there were no differences between groups (OR 0.48; 95% CI 0.19–1.23; I2 = 40%; Supplementary Figure S6).

#### Severe compensatory sweating

In the subgroup analysis by study design, the reduction in severe CS associated with T2-sparing sympathectomy was observed in both the non-RCT subgroup (OR 0.41; 95% CI 0.24–0.72; I2 = 49%) and the RCT subgroup (OR 0.41; 95% CI 0.17–0.98; I2 = 27%). There was no significant difference between these subgroups (p = 0.9703), as shown in Supplementary Figure S7.

In the subgroup analysis by follow-up duration, the reduction in severe CS associated with T2-sparing sympathectomy was observed at both > 12 months (OR 0.29; 95% CI 0.18–0.48; I^2^ = 0%) and ≤ 12 months (OR 0.59; 95% CI 0.37–0.94; I^2^ = 0%). There was a significant difference between these subgroups (p = 0.0435), as shown in Supplementary Figure S8.

In the subgroup analysis by surgical technique, the reduction in severe CS associated with T2-sparing sympathectomy was observed in both the cautery subgroup (OR 0.41; 95% CI 0.17–0.98; I2 = 27%) and the clipping subgroup (OR 0.41; 95% CI 0.24–0.72; I2 = 49%). There was no significant difference between these subgroups (p = 0.9703), as shown in Supplementary Figure S9.

### Sensitivity analysis

Leave-one-out sensitivity analyses were conducted to assess the influence of individual studies on the pooled estimates. For overall CS, excluding any single study shifted the pooled result from significant (favoring the T2-sparing group) to non-significant, indicating sensitivity to individual studies; notably, excluding the study by Esme et al. [21] eliminated the observed heterogeneity without altering the direction of the overall effect (Supplementary Figure S1).

### Risk of bias assessment

Four randomized controlled trials were evaluated using the RoB 2 tool (Supplementary Table II). One trial was judged to have a low risk of bias across all domains [18]. The second trial raised some concerns related to selective outcome reporting [23]. The third trial was rated as having high risk of bias due to insufficient clarity in the randomization process and a high risk of reporting bias [27]. The fourth trial was judged to have some concerns overall, primarily due to limited reporting of the randomization process, potential bias in subjective outcome measurement, and the absence of a prespecified protocol [28].

Seven observational studies were assessed using the ROBINS-I tool (Supplementary Table III). Most of these studies were judged to have a serious overall risk of bias, primarily due to inadequate control of confounding factors and reliance on unvalidated, self-reported outcome measures. Two studies were classified as having a critical risk of bias, largely driven by missing outcome data and methodological limitations in outcome assessment [22, 25]. Although these methodological concerns may have affected individual study quality; however, the direction and magnitude of effect estimates remained consistent across studies. Leave-one-out sensitivity analyses confirmed the robustness of the primary results.

Assessment of publication bias revealed no major concerns. The funnel plot for CS showed a relatively symmetrical distribution of studies around the pooled effect estimate (Supplementary Figure S10). This was further supported by Egger’s test (t = – 0.18; df = 8; p = 0.86) and Begg’s test (z = – 0.09; p = 0.93), indicating no statistically significant evidence of small-study effects. These findings suggest a low likelihood of substantial publication bias.

## Discussion

In this systematic review and meta-analysis of eleven studies comprising 3,090 patients with primary hyperhidrosis undergoing thoracic sympathectomy, we found that T2‑sparing sympathectomy was associated with a significantly lower incidence of both overall and severe CS. A leave‑one‑out sensitivity analysis showed that excluding the study by Esme et al. removed heterogeneity without changing the direction of the effect, underscoring the robustness of our findings.

The protective effect of the T2-sparing approach may be attributed to the partial preservation of sympathetic fibers involved in sudomotor control, which helps mitigate the compensatory increase in sweating in untreated regions [29]. While earlier preclinical studies and small clinical series had previously suggested this association, our meta-analysis reinforces these findings within a larger and statistically robust cohort [24, 29]. This is consistent with previous systematic reviews and meta-analyses showing that sympathectomy performed at lower levels, particularly at T3 or T4 while sparing T2, is associated with a significantly reduced incidence and severity of CS, without compromising the therapeutic effectiveness for palmar hyperhidrosis [8, 30, 31].

When stratified by study design, the reduction in CS remained consistent across both RCTs and observational studies, with no statistically significant differences observed between these subgroups. This methodological concordance reinforces the external validity of T2-sparing sympathectomy, suggesting that the observed benefit is not merely a function of study design or bias. The convergence of evidence from multiple sources, indicates that sparing the T2 ganglion effectively reduces CS in different clinical settings and populations, thereby supporting its generalizability and applicability in routine surgical practice [20, 27, 32].

Moreover, the pronounced decrease in CS was observed not only in the immediate postoperative period but also at intermediate and extended follow-up intervals, demonstrating that the advantages of sparing the T2 ganglion extend well beyond the early recovery phase. This enduring effect implies a durable rebalancing of sudomotor function, translating into sustained symptom relief and high patient satisfaction over time. Such consistency across diverse postoperative time points strengthens the case for T2-sparing sympathectomy as a reliable, long-term solution for managing palmar hyperhidrosis with minimal late-onset complications [7, 33].

Real-world applicability of our results is underscored by the Brazilian retrospective cohort study by Wolosker et al., which evaluated 106 patients undergoing thoracoscopic sympathectomy with and without T2 preservation [34]. In that series, the T2-sparing group exhibited a significantly reduced incidence of compensatory hyperhidrosis (50% vs. 72%; p = 0.019) without compromising palmar sweating resolution, mirroring the pooled effect size in our meta-analysis [34]. Crucially, these findings demonstrate that the protective benefit of T2 preservation transcends controlled trial settings and remains robust across diverse healthcare environments, including middle-income countries, thereby confirming the external validity and practical value of T2-sparing VATS sympathectomy in everyday clinical practice [32, 35].

In our exploratory analysis, the benefit of T2-sparing sympathectomy remained evident in studies in which both groups had the same number of interrupted levels, suggesting that the observed reduction in compensatory sweating cannot be explained solely by a less extensive denervation in the T2-sparing arm. In contrast, among studies in which the T2-sparing group underwent interruption of fewer total levels than the control group, this difference was no longer statistically significant [23, 30]. These results suggest that compensatory sweating may be influenced by both T2 involvement and the overall extent of denervation [36–38]. These findings suggest that surgical planning should consider not only whether T2 is preserved, but also the overall extent of denervation, with the goal of achieving symptom control through the least extensive interruption possible [19, 31].

This study has limitations. First, the majority of the included studies were observational, introducing potential selection bias. To address this, subgroup analyses were performed based on study design, but we cannot completely rule out residual confounding. Second, the definitions of CS and its severity varied across studies. Although similar criteria were used, the lack of standardized outcome definitions may have influenced results. Third, the moderate heterogeneity observed for overall CS suggests there were methodological or population differences, despite sensitivity analyses confirming the direction of effect. Fourth, relatively few studies included follow-up beyond 12 months, limiting assessment of long-term outcomes. Fifth, this meta-analysis was restricted to compensatory sweating and did not directly assess whether T2-sparing approaches provide equivalent control of the primary hyperhidrosis symptoms; the decision to spare T2 should therefore also weigh any potential trade-off in primary symptom control. In addition, although our exploratory analysis suggested that the benefit of sparing T2 was not attributable solely to a less extensive denervation, this comparison was based on few studies with wide confidence intervals and substantial heterogeneity, so the individual contributions of T2 preservation and of the overall extent of denervation cannot be fully disentangled. Finally, given the predominance of observational data and the serious-to-critical risk of bias observed in several included studies, the overall certainty of the evidence should be regarded as limited.

Risk of bias assessments revealed varying methodological quality among the included studies. While randomized trials generally exhibited a low or moderate risk of bias, most observational studies were rated as having a serious or critical risk, particularly due to inadequate control of confounding factors and the reliance on self-reported outcomes without standardized validation. Nonetheless, effect estimates remained consistent across studies with different risk profiles, and leave-one-out sensitivity analyses confirmed the robustness of the main findings. Furthermore, visual inspection of funnel plots and formal statistical tests (Egger’s and Begg’s) did not identify significant small-study effects, suggesting a low likelihood of publication bias.

Despite these limitations, our review adhered strictly to PRISMA guidelines and utilized validated tools for risk of bias assessment, ensuring methodological rigor and transparency throughout the process. From an initial pool of 433 records, only 11 studies met all predefined inclusion criteria, comprising exclusively comparative analyses of T2-including versus T2-sparing sympathectomy. This rigorous and reproducible selection process strengthens the internal validity of our findings and supports the reliability of the pooled estimates. Given that thoracic sympathectomy is typically an elective procedure performed on otherwise healthy, predominantly young individuals, reducing postoperative morbidity, particularly the incidence of CS, is of critical clinical importance. Our results indicate that T2-sparing sympathectomy should be considered a preferred surgical strategy when anatomically and technically feasible.

## Conclusion

This meta-analysis found that T2-sparing sympathectomy is associated with a significantly lower occurrence of CS compared to T2-including procedures. These results were consistent across various study designs and remained robust in sensitivity analyses. Given the elective nature of sympathectomy, especially in younger patients, preserving the T2 level may help reduce postoperative morbidity. Further prospective studies are needed to confirm these findings and guide surgical decision-making.

## Supplementary Information

Supplementary material 1.

## Data Availability

No datasets were generated or analysed during the current study.
