# Supplementary material for: T2-sparing vs T2-including sympathectomy for hyperhidrosis: a meta-analysis on compensatory sweating
Source: J Cardiothorac Surg. 2026 Jun 25;21:445. doi: 10.1186/s13019-026-04464-4 (PMC13307732; doi:10.1186/s13019-026-04464-4)

**SUPPLEMENTARY MATERIAL**

**TITLE:** T2-Sparing vs T2-Including Sympathectomy for Hyperhidrosis: A Meta-Analysis on Compensatory Sweating

**SUPPLEMENTARY TABLES AND FIGURES LEGENDS:**

**Supplementary Table I.** Definitions and assessment methods of compensatory sweating.

**Supplementary Table II.** Risk of bias assessment for randomized controlled trials.

**Supplementary Table III.** Risk of bias assessment for observational studies.

**Supplementary Figure S1.** Leave-one-out analysis for compensatory sweating. OR: odds ratio; CI: confidence interval.

**Supplementary Figure S2.** Subgroup analysis for overall compensatory sweating based on study design.

**Supplementary Figure S3.** Subgroup analysis for overall compensatory sweating based on follow-up duration.

**Supplementary Figure S4.** Subgroup analysis for overall compensatory sweating based on surgical technique.

**Supplementary Figure S5.** Subgroup analysis for overall compensatory sweating based on denervation extent: same number of interrupted levels in both arms.

**Supplementary Figure S6.** Subgroup analysis for overall compensatory sweating based on denervation extent: fewer interrupted levels in the T2-sparing group than in the control group.

**Supplementary Figure S7.** Subgroup analysis for severe compensatory sweating based on study design.

**Supplementary Figure S8.** Subgroup analysis for severe compensatory sweating based on follow-up duration.

**Supplementary Figure S9.** Subgroup analysis for severe compensatory sweating based on surgical technique.

**Supplementary Figure S10.** Funnel plot for overall compensatory sweating.

**Supplementary Table I.** Definitions and assessment methods of compensatory sweating.

| **Study** | **Definition of CS** | **Definition of Severe CS** | **Type of Evaluation** | **Assessment Method** | **Follow-up** | **Specific Measurement Details** |
| --- | --- | --- | --- | --- | --- | --- |
| Cai, 2014 | Defined as postoperative sweating in areas not previously affected or less affected | Defined as sweating that is embarrassing or interferes with daily activities | Categorical | Structured questionnaire | 1, 6, and 12 months | Graded classification (mild, moderate, severe) |
| Liu, 2004 | Defined as postoperative sweating in other body regions after sympathectomy | Defined as sweating requiring frequent clothing changes or discomfort | Functional / Categorical hybrid | Patient-reported questionnaire | Not clearly specified | Functional criteria; Reisfeld-type |
| Chang, 2007 | Defined as sweating in less affected areas postoperatively | NR | Continuous | Telephone questionnaire (VAS 0–10) | ~47 months | Visual Analog Scale (VAS) |
| Ersin, 2024 | Reported as presence of postoperative sweating | NR | Binary | Retrospective + telephone | 4–77 months | Dichotomous (yes/no) + anatomical distribution |
| Esme, 2019 | Defined via patient report | NR | Categorical (scale-based) | Telephone + clinical | ~13 months | HDSS (validated 4-point scale) |
| Reisfeld, 2007 | Defined as sweating in new areas | Socially embarrassing / interferes with life | Categorical (functional) | Patient-reported | Not clearly specified | Functional classification (mild/moderate/severe) |
| Salim, 2018 | Defined as new excessive sweating after surgery | Interferes with daily activities | Categorical (functional grading) | Clinical + telephone | 1, 6, and 12 months | Explicit mild/moderate/severe classification |
| Schmidt, 2006 | Defined as increased sweating in new areas | NR | Categorical (subjective) | Questionnaire | Not clearly specified | No standardized scale; subjective perception |
| Scognamillo, 2011 | Described as postoperative sweating occurring in other body regions after sympathectomy | Not formally defined; impact inferred from effect on quality of life | Continuous (QoL-based) / Subjective | Questionnaire + telephone follow-up | 1–15 years | Uses numerical QoL impact scale (1–10) assessing domains (social, work, affectivity, activities); explicitly states that CS is difficult to define and quantify |
| Sugimura, 2009 | Defined as patient-reported postoperative sweating in other body regions after sympathectomy | Defined as VAS score 8–10 | Continuous → Categorized (threshold-based) | Prospective assessment using Visual Analog Scale (VAS 0–10) | Median 10.4 months (range 0–83 months) | Uses VAS for both satisfaction and CS; explicitly categorizes severe CS using a numeric cutoff (8–10), allowing reproducibility |
| Turkyilmaz, 2017 | Described as postoperative sweating in previously unaffected areas (back, abdomen, groin, legs) | Described qualitatively as “severely irritating” and associated with patient dissatisfaction | Subjective / Clinical (non-standardized categorical) | Clinical interviews + physical examination | 1 month and 12 months | No validated scale; severity inferred from patient dissatisfaction and impact on daily life; authors explicitly acknowledge need for validated objective criteria |
| Yazbek, 2009 | Defined as new-onset sweating in areas not previously affected after sympathectomy | Defined as sweating that is intolerable or interferes with daily activities, based on patient report | Subjective / Functional categorical | Structured patient questionnaire | Not clearly standardized (variable follow-up reported) | Severity based on impact on quality of life and tolerance; no validated scale (e.g., HDSS), but uses functional impairment criteria |
| **Abbreviations:** CS, compensatory sweating; HDSS, Hyperhidrosis Disease Severity Scale; VAS, visual analog scale; QoL, quality of life; NR, not reported. | | | | | | |

**Supplementary Table II.** Risk of bias assessment for randomized controlled trials.

| **Study** | **Bias from randomization process** | **Bias due to deviations from intended interventions** | **Bias due to missing outcome data** | **Bias in measurement of the outcomes** | **Bias in selection of the reported result** | **Overall risk of bias** |
| --- | --- | --- | --- | --- | --- | --- |
| Cai, 2014 | Low | Low | Low | Low | Low | Low |
| Salim, 2018 | Low | Low | Low | Low | Some concerns | Some concerns |
| Turkyilmaz, 2017 | Some concerns | Low | Low | Low | High | High |
| Yazbek, 2009 | Some concerns | Low | Low | Some concerns | Some concerns | Some concerns |

Sterne JAC, Savović J, Page MJ, Elbers RG, Blencowe NS, Boutron I, Cates CJ, Cheng H-Y, Corbett MS, Eldridge SM, Hernán MA, Hopewell S, Hróbjartsson A, Junqueira DR, Jüni P, Kirkham JJ, Lasserson T, Li T, McAleenan A, Reeves BC, Shepperd S, Shrier I, Stewart LA, Tilling K, White IR, Whiting PF, Higgins JPT. RoB 2: a revised tool for assessing risk of bias in randomised trials. *BMJ* 2019; **366**: l4898.

**Supplementary Table III.** Risk of bias assessment for observational studies.

| **Study** | **Bias due to confounding** | **Bias in selection of participants** | **Bias in classification of interventions** | **Bias due to deviations from intended interventions** | **Bias due to missing data** | **Bias in measurement of outcomes** | **Bias in selection of the reported result** | **Overall risk of bias judgement** |
| --- | --- | --- | --- | --- | --- | --- | --- | --- |
| Chang 2007 | Moderate | Serious | Low | Moderate | Serious | Serious | Low | Serious |
| Ersin 2014 | Moderate | Low | Low | Low | Low | Moderate | Low | Moderate |
| Esme 2019 | Moderate | Moderate | Low | Low | Moderate | Serious | Low | Serious |
| Reisfeld 2007 | Serious | Serious | Low | Low | Critical | Serious | Low | Critical |
| Schmidt 2006 | Serious | Moderate | Low | Low | Moderate | Moderate | Low | Serious |
| Sugimura 2009 | Serious | Moderate | Low | Low | Moderate | Serious | Low | Serious |
| Scognamillo 2011 | Serious | Moderate | Low | Low | Critical | Moderate | Low | Critical |

ROBINS-I tool: Sterne et al. BMJ 2016;355:i4919.

**Supplementary Figure S1.** Leave-one-out analysis for compensatory sweating. OR: odds ratio; CI: confidence interval.


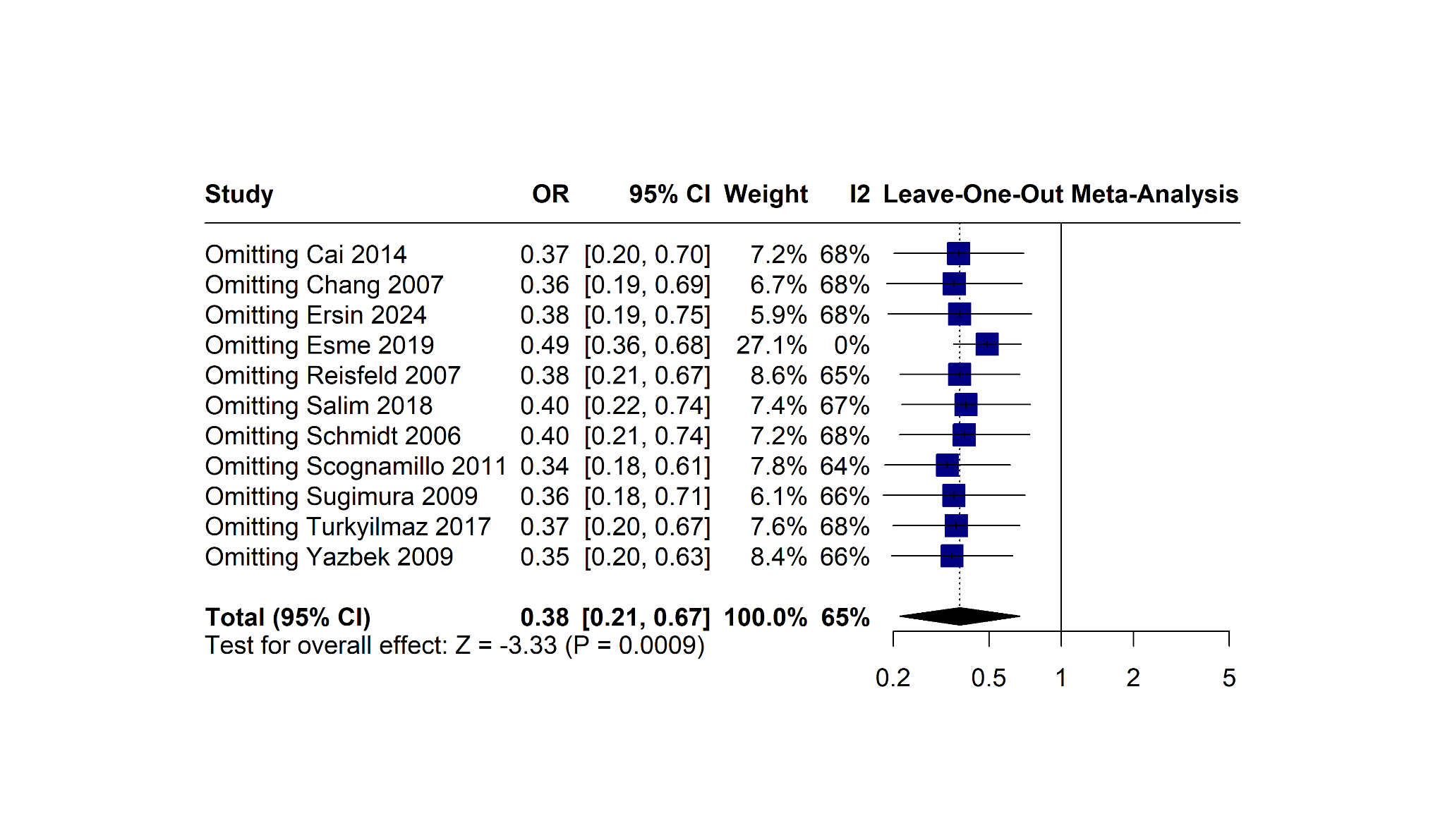


**Supplementary Figure S2.** Subgroup analysis for overall compensatory sweating based on study design.


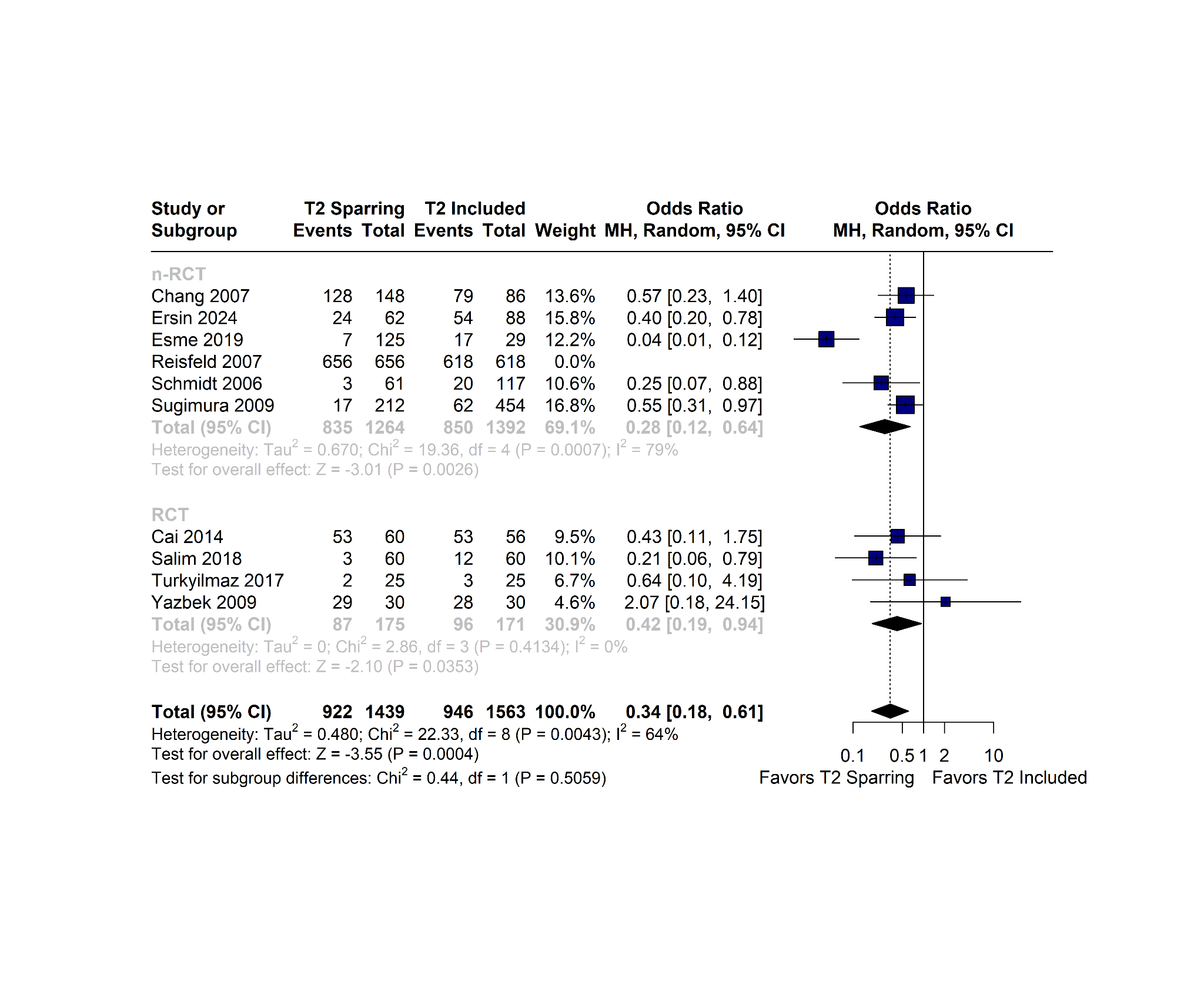


**Supplementary Figure S3.** Subgroup analysis for overall compensatory sweating based on follow-up duration.


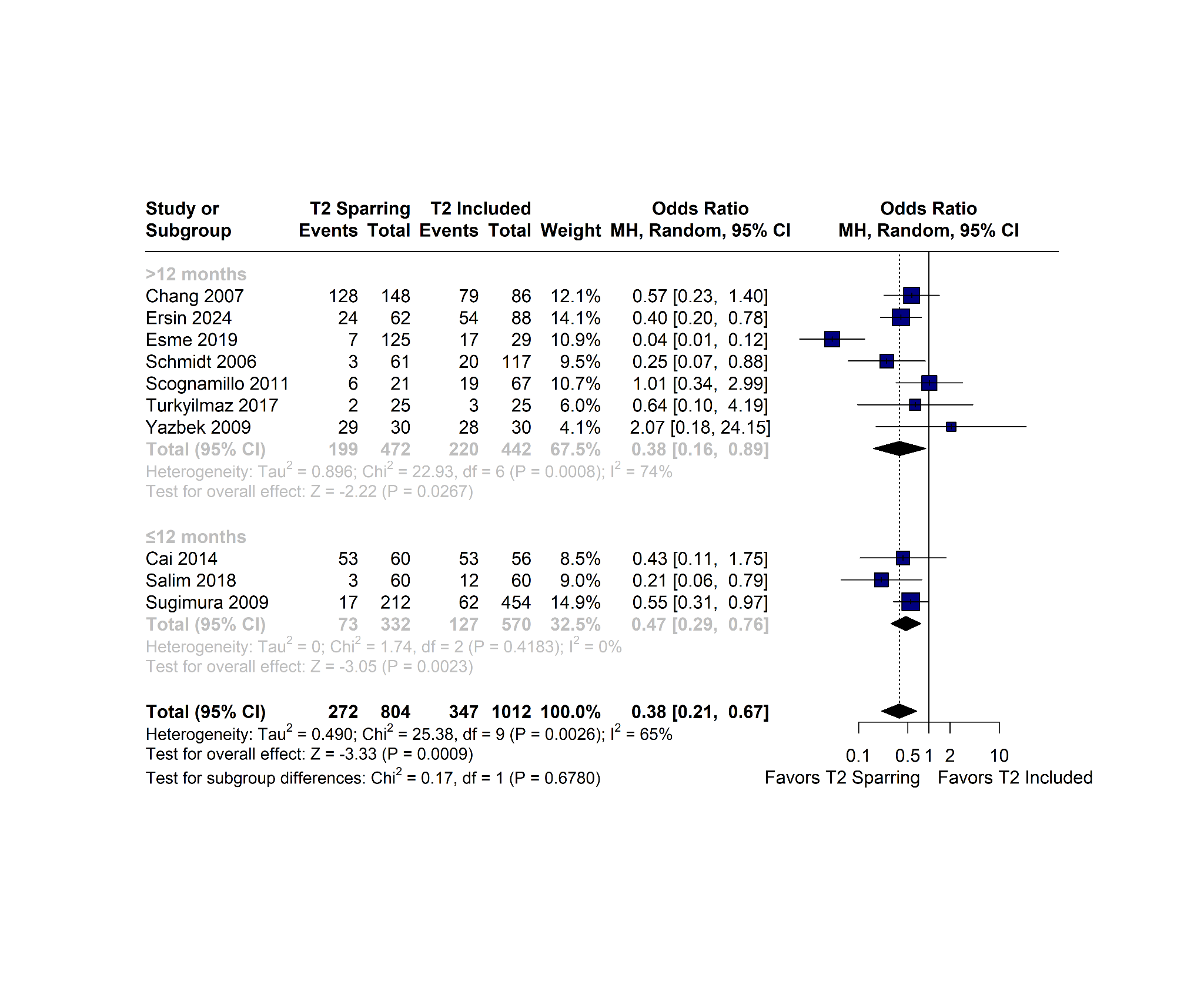


**Supplementary Figure S4.** Subgroup analysis for overall compensatory sweating based on surgical technique.


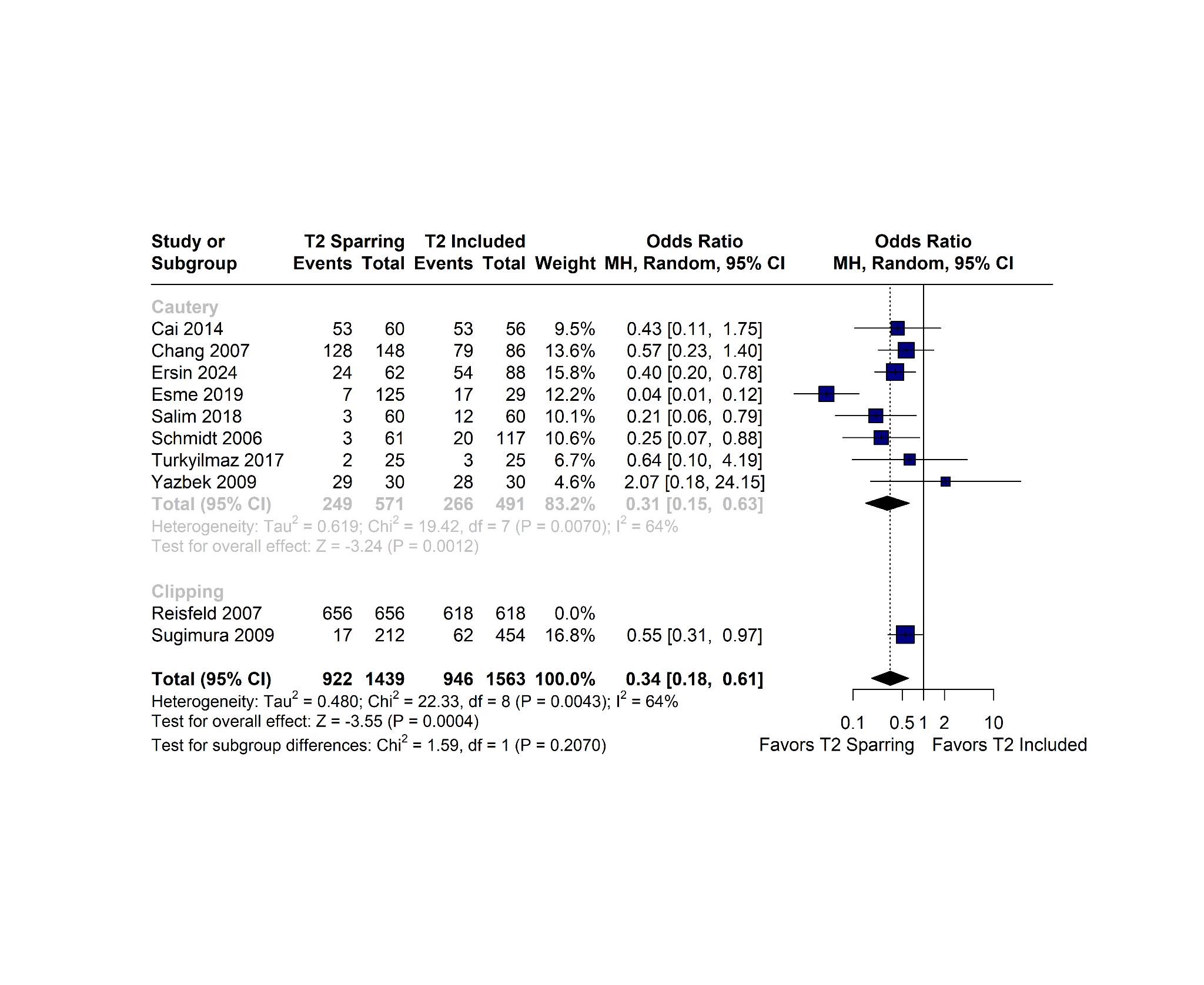


**Supplementary Figure S5.** Subgroup analysis for overall compensatory sweating based on denervation extent: same number of interrupted levels in both arms.


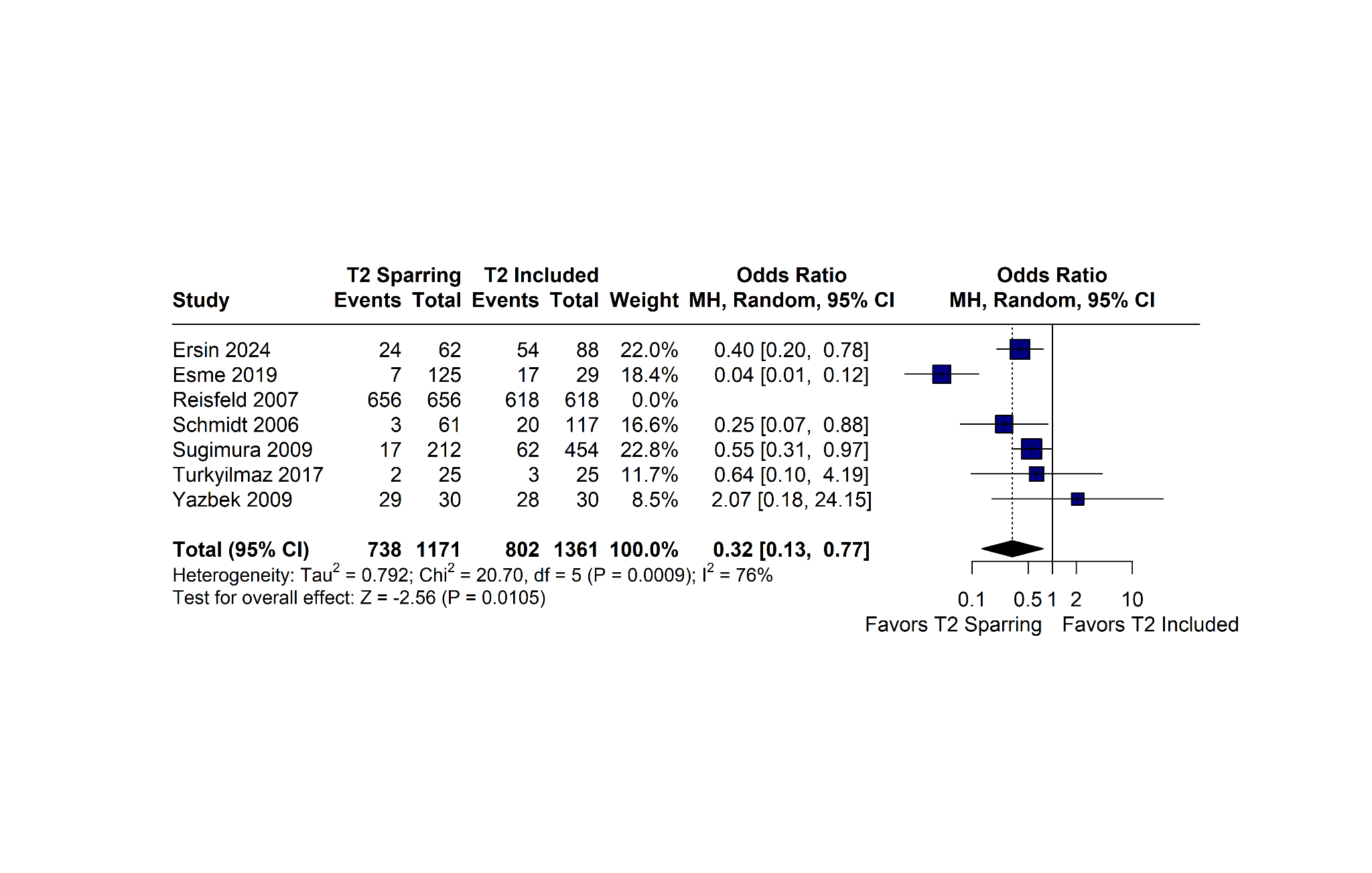


**Supplementary Figure S6.** Subgroup analysis for overall compensatory sweating based on denervation extent: fewer interrupted levels in the T2-sparing group than in the control group.


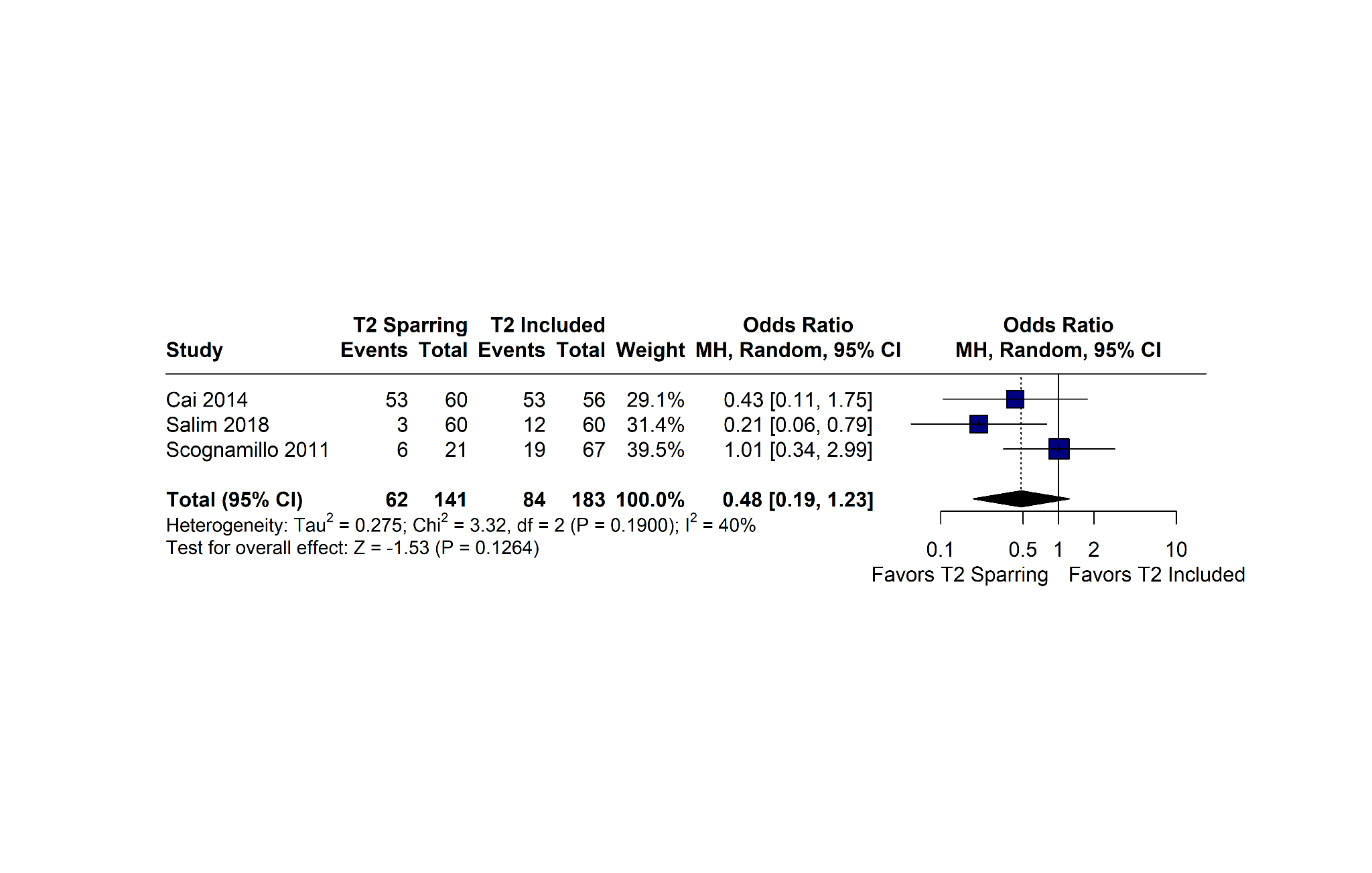


**Supplementary Figure S7.** Subgroup analysis for severe compensatory sweating based on study design.


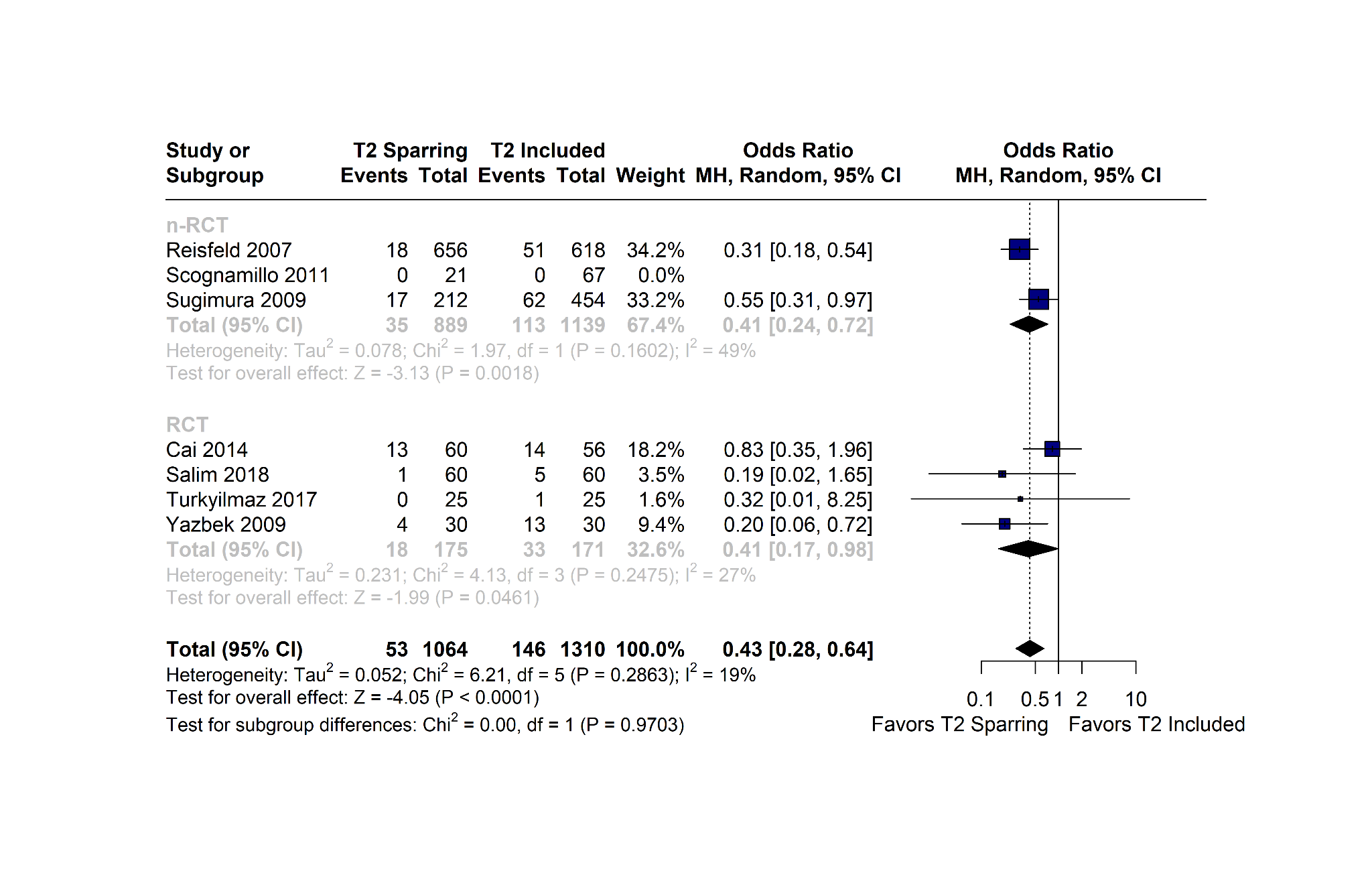


**Supplementary Figure S8.** Subgroup analysis for severe compensatory sweating based on follow-up duration.


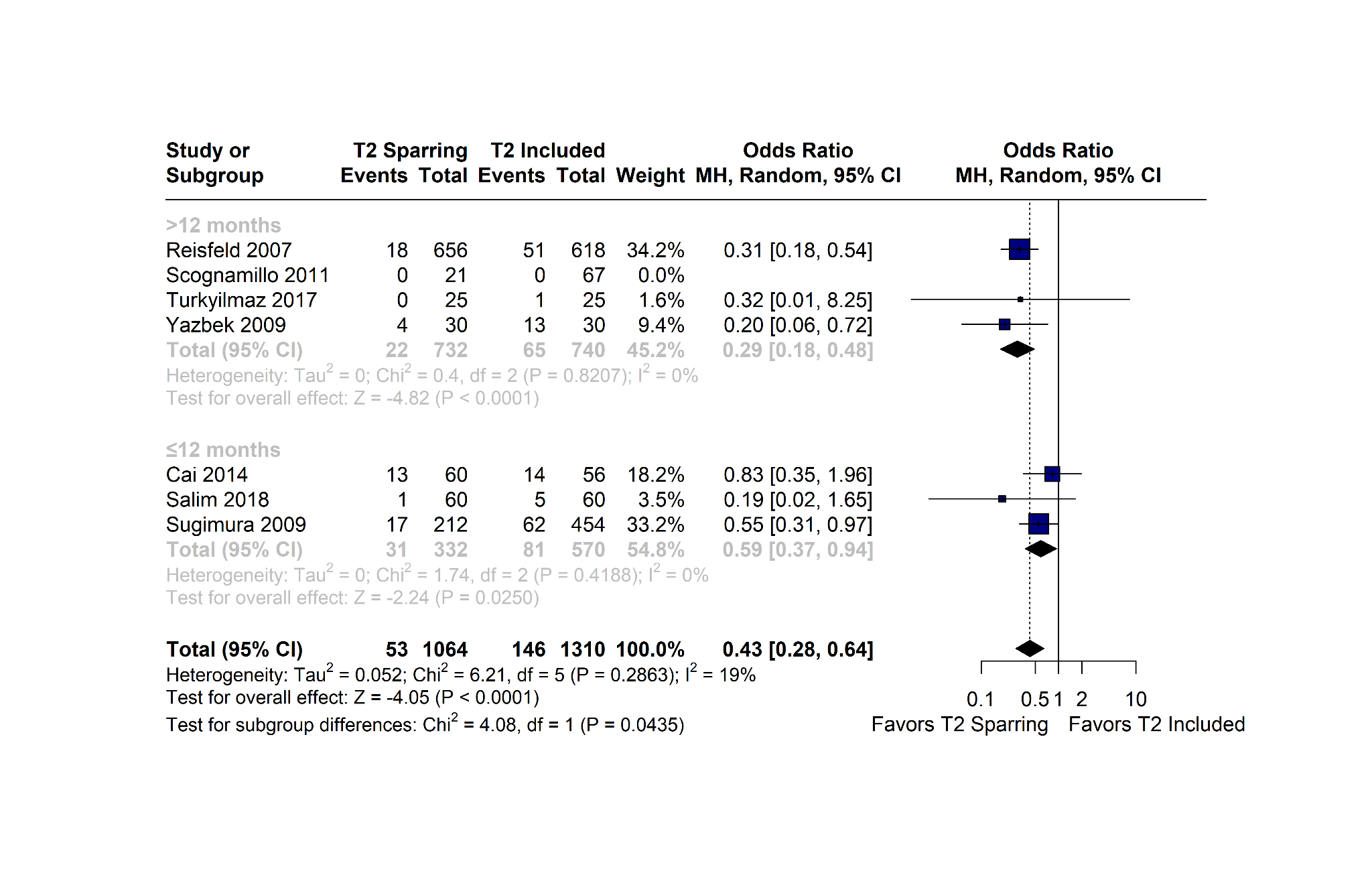


**Supplementary Figure S9.** Subgroup analysis for severe compensatory sweating based on surgical technique.


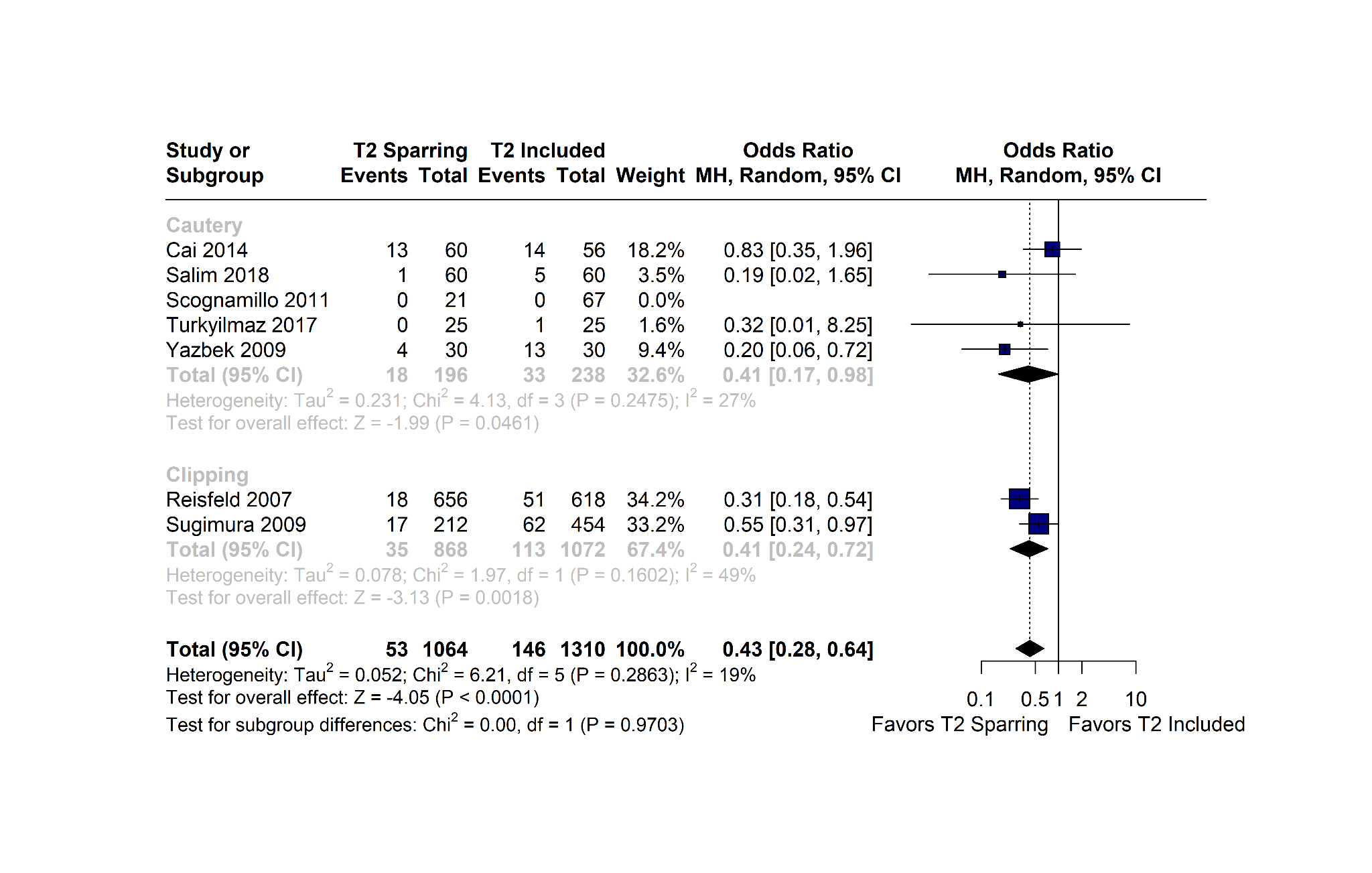


**Supplementary Figure S10.** Funnel plot for overall compensatory sweating.


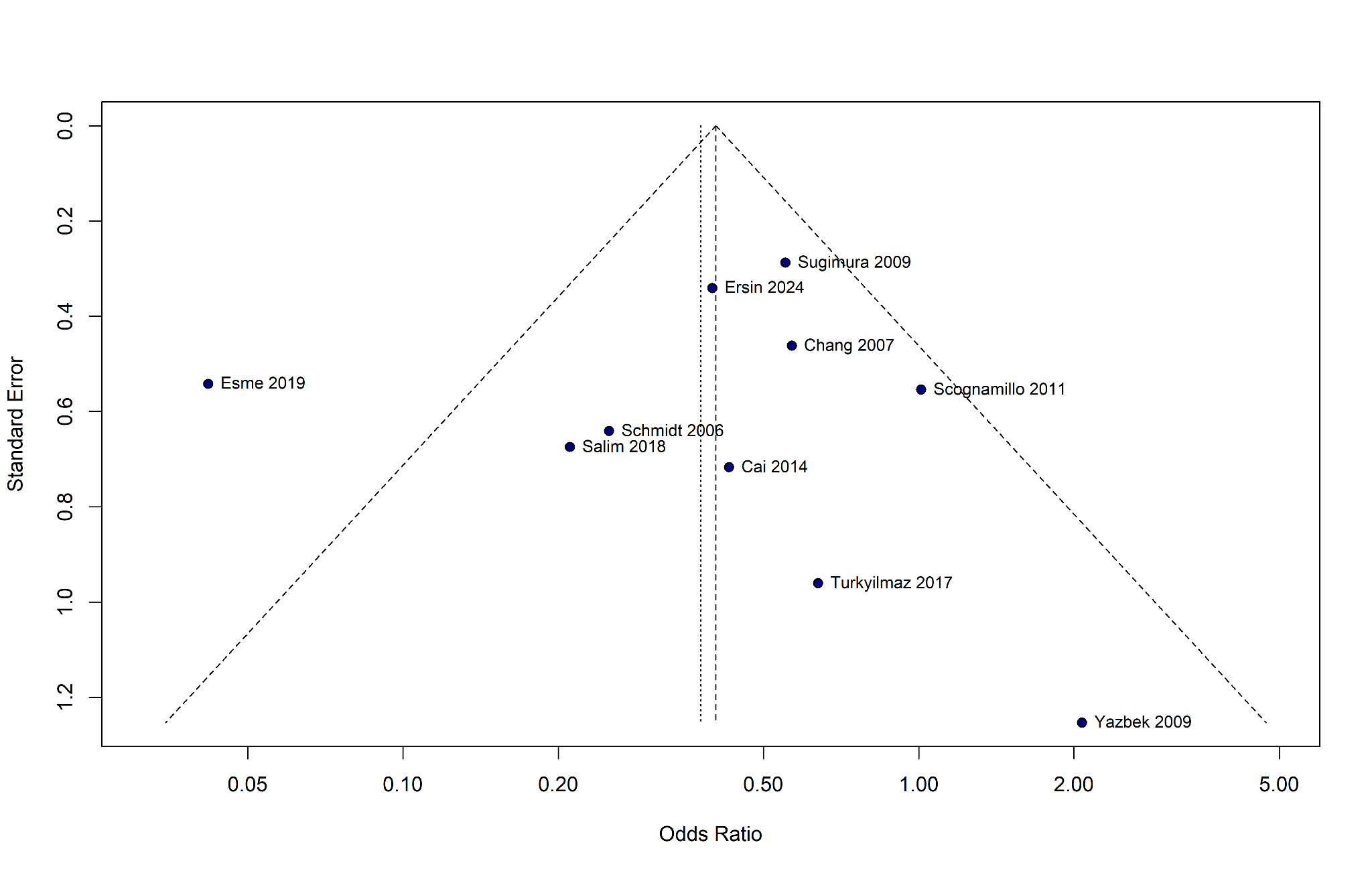

Supplement: Supplementary file 1 — Supplementary material 1. [file 13019_2026_4464_MOESM1_ESM.docx]
